# Evaluation of Plasma Phosphorylated Tau217 for Differentiating Alzheimer's Disease

**DOI:** 10.1002/alz70856_103744

**Published:** 2025-12-26

**Authors:** Anna Mammel, Ging‐Yuek Robin Hsiung, Ali Mousavi, Pankaj Kumar, Kelsey Hallett, Ian R MacKenzie, Veronica Hirsch‐Reinshagen, Donald Biehl, Pradip Gil, Mary Encarnacion, Hans Frykman

**Affiliations:** ^1^ Neurocode USA Inc., Bellingham, WA, USA; ^2^ University of British Columbia, Vancouver, BC, Canada; ^3^ Division of Neurology‐ Department of Medicine‐ University of British Columbia, vancouver, BC, Canada; ^4^ BC Neuroimmunology Lab, Vancouver, BC, Canada; ^5^ The University of British Columbia, Vancouver, BC, Canada; ^6^ Neurocode Lab, Bellingham /WA, WA, USA; ^7^ BC Neuroimmunology lab, Vancouver, BC, Canada; ^8^ Division of Neurology‐ Department of Medicine, University of British Columbia, Vancouver, Vancouver, BC, Canada

## Abstract

**Background:**

Blood‐based biomarkers provide an accessible and cost‐effective tool for diagnosing Alzheimer's disease (AD). Among these biomarkers, phosphorylated tau 217 (*p*‐tau217) shows promise for AD diagnosis, differential diagnosis, and facilitating access to emerging disease‐modifying therapies. This study evaluates the ALZpath plasma *p*‐tau217 assay as a Laboratory Developed Test for distinguishing AD pathology from other concurrent pathologies associated with AD.

**Method:**

We examined EDTA plasma samples of autopsy confirmed (*n* = 110) of patients assessed at the UBC Hospital Clinic for Alzheimer's disease. The cohort was characterized as AD only (*n* = 30), AD with CAA (*n* = 7), AD with DLB (*n* = 13), AD with Vascular (*n* = 7), AD with mix pathologies (*n* =  13), TDP‐43 only (*n* =  18), Synuclein only(*n* = 4), Tau only (*n* = 18). The samples were analyzed by the ALZpath *p*‐tau217 v2 assay on a single Quanterix HD‐X SIMOA Analyzer platform.

**Results:**

While the plasma *p*‐tau217 concentrations in cases with AD (1.6 ± 0.8 ng/L) were higher than those in AD with DLB cases (1.1 ± 0.7 ng/L) and cases with AD plus vascular pathology (1.0 ± 0.6 ng/L), and lower than in cases with AD plus CAA (1.7 ± 0.6 ng/L), these differences were not statistically significant. However, the concentrations were significantly higher than those in cases with AD with mixed pathologies (0.7 ± 0.4 ng/L), TDP‐43 only (0.3 ± 0.2 ng/L), synuclein only (0.4 ± 0.1 ng/L), and tau only (0.3 ± 0.2 ng/L) (*p* < 0.001).

**Conclusion:**

In our current study, plasma *p*‐tau217 levels did not differentiate AD with DLB, AD with vascular pathology, or AD with CAA cases from the AD group, though they could differentiate AD from cases with mixed pathologies, TDP‐43 only, synuclein only, and tau‐only pathology. This suggests co‐existing AD pathology, making their *p*‐tau measurements similar to the AD group. Further studies with autopsy‐defined samples will be needed to clarify the role of plasma *p*‐tau217 in these groups.

